# Antimicrobial Efficacy Assessment of Human Derived Composite Amnion-Chorion Membrane

**DOI:** 10.1038/s41598-019-52150-4

**Published:** 2019-10-30

**Authors:** Nathan D. Palanker, Chun-Teh Lee, Robin L. Weltman, Gena D. Tribble, Ransome van der Hoeven, Jianming Hong, Bingyan Wang

**Affiliations:** The University of Texas Health Science Center at Houston School of Dentistry, Houston, Texas 77054 USA

**Keywords:** Antimicrobials, Translational research

## Abstract

Human derived composite amnion-chorion membrane (ACM) has been used to facilitate wound healing due to reported anti-inflammatory properties and promotion of cell proliferation. This study aimed to assess the antimicrobial properties of the ACM using novel methods to visualize the antimicrobial efficacy of membranes *in situ* at different time points. Porcine Pericardium Collagen Membranes (PPCM) served as membrane controls. Circular pieces of the membranes were used in three different assays: insert, agar contact and glass-bottom well assays. *Streptococcus gordonii* were spotted onto the membranes and the plates were subsequently centrifuged to ensure direct bacterial contact with the membranes in the insert and agar contact assays, thus better mimicking bacterial adherence in the oral cavity. After incubation at 37 °C for 8, 24, and 48 hours, the membranes were dyed with the Live/Dead BacLight Bacterial Viability fluorescence stain and analyzed via confocal microscopy. The results demonstrated that the ACM completely inhibited bacterial growth at all time points, whereas the PPCM did not demonstrate any antimicrobial properties. Within the limits of this study, the ACM showed extremely high antimicrobial efficacy against oral streptococci. In addition, our methods may be useful in assessing antimicrobial properties for biomaterials with minimum diffusion ability, when traditional assessment methods are not applicable.

## Introduction

Resorbable and non-resorbable barrier membranes, comprised of natural or synthetic biocompatible materials, are widely used for guided tissue regeneration and guided bone regeneration in dentistry due to their clinical predictability and reliability. However, membrane exposure during wound healing is one of the major complications that may compromise clinical outcomes^1,2^, resulting in failure to regenerate lost periodontal structures. Early membrane exposure gives rise to loss of material integrity and microbial contamination^1,3,4^. Since bacterial colonization in the surgical site is a common clinical problem, some membranes were developed or modified to contain antimicrobial properties. A study by Barber *et al*. claimed that a high-density PTFE membrane with a small pore size (0.2 µm), smaller than the size of a bacterial cell, could prevent infiltration of bacteria and thus enhance regeneration of bone and soft tissue in surgical sites^5^. However, the adherence of bacteria to the membrane may still compromise clinical results if primary closure is not achieved. Antibiotics were added in the expanded PTFE and collagen membranes to increase antibacterial efficacy^6–8^. However, some patients may be allergic to antibiotics or may develop antibiotic resistance^9^. Additionally, it is challenging to maintain the minimum inhibitory concentrations of the antibiotics in the oral cavity during the wound healing phase due to the continual secretion of saliva^10,11^.

A human derived composite amnion-chorion membrane (ACM) is made from donated human placentae. The amnion-chorion tissue of the placenta is processed to cleanse and maintain the delicate structures of the tissue^12^. The processing methodology allows for retention of the biological structures and molecules found in native amnion-chorion tissue. The ACM has been used to facilitate wound healing, for diabetic ulcers^13^, chronic wounds^14^, perforated sinus membranes^15^ and alveolar socket preservation^16^, because this processed placental tissue-derived membrane retains extracellular matrix proteins, growth factors and cytokines that promote cell proliferation and control inflammation at the wound site^17,18^. Additionally, the ACM may have antimicrobial properties as demonstrated through studies showing that cells of human amniotic and chorion membranes origins produce antimicrobial peptides^19,20^. However, the antimicrobial properties have never been assessed and demonstrated in any processed placental tissue-derived product.

Historically, the antimicrobial activity of biomaterials has been tested on agar plates^21^. Inhibition of bacterial growth creates a zone void of bacterial colonies around the tested antimicrobial disks or membranes. This method, known as the agar diffusion assay or disc diffusion assay, requires diffusion of antimicrobial components into the agar and is suitable for assessing membranes with added antibiotics or medications. However, it is not suitable for assessing materials that do not easily diffuse or release antimicrobial components into the agar. In addition, it is not possible to assess the antimicrobial activity of membranes at different time points or to observe the bacterial growing on the membranes *in situ*. Since the ACM is a preserved and processed allogenic tissue membrane, it is not expected to easily observe proactive release of biological molecules as can be seen in living tissues. However, the ACM may still maintain antimicrobial peptides or other components that can inhibit the growth of bacteria when bacteria attach to the membrane. Therefore, a new method is required to properly assess the potential antimicrobial property of the ACM and other processed or synthetic membranes without added antibiotics or medications that can be easily released. Additionally, a new method should be able to simulate the adherence of bacterial cells to the biomaterial and to assess the efficacy of antimicrobial activity at different time points, which cannot be demonstrated in the agar diffusion assay.

This study assessed antimicrobial properties of ACM by three novel methods: insert assay, agar contact assay, and glass-bottom well assay. The inhibition of bacterial growth on membranes was directly assessed on the interface between bacterial cells and the membrane using confocal microscopy. This is the first study using these novel assays to assess antimicrobial activities of clinically used bio-membranes.

## Methods

### Amnion-chorion membranes

A human derived composite amnion-chorion membrane (ACM, BioXclude®, Snoasis Medical, CO, USA) is a dehydrated collagenous allograft comprised of amnion and chorion layers derived from the human placenta. Human placentas were donated under informed consent as regulated by the Food and Drug Administration’s (FDA) Good Tissue Practice and American Association of Tissue Banks (AATB). All donors were tested to be free of infectious diseases, including human immunodeficiency virus (HIV), human T-cell lymphotropic virus (HTLV), Hepatitis B and C, syphilis, and Cytomegalovirus (CMV)^22^. The amniotic and chorionic membranes were processed using a proprietary PURION® process that involves gentle cleansing and dehydrating the membranes while preserving the structural integrity and biochemical activity of the tissue. The amniotic membrane and the chorionic membrane were either processed separately, or laminated to form a two-layer graft. The composite amnion-chorion membrane is significantly thicker (~300 μm) compared to the layered amnion alone (<100 μm). Processed ACMs are terminally sterilized by gamma irradiation, which has been proven not to affect the bioactivity of the allograft, prior to sterile packaging^23^. Commercially available ACMs were obtained from Snoasis Medical and used directly in this study.

### Porcine pericardium collagen membranes

Porcine Pericardium Collagen Membranes (PPCM; Vitala®, Osteogenics Biomedical Inc., Lubbock, TX, USA) are membranes manufactured using a proprietary process designed to maintain a microporous, 3-layered tissue architecture (parietal pericardium, visceral pericardium and fibrous pericardium) without the need for cross-linking chemicals and agents. The natural 3-layered architecture provides a high tensile strength and clinical adaptation. PPCMs were used as the control membranes in this study.

### Bacteria strains and media

*Streptococcus gordonii* Challis, a Gram-positive bacterium and one of the initial colonizers in the oral cavity, was used as a representative oral early colonizer in this study. *S*. *gordonii* was inoculated on Todd Hewitt agar plates [composed of 3 g of dehydrated Todd Hewitt Broth (THB, Difco, Detroit, MI, USA) and 1.5 g agar in 100 ml dH_2_O], and cultured for 36–48 hours at 37 °C in an anaerobic jar containing a bag of BD BBL CO_2_ generator (Becton, Dickinson and Company, Sparks, MD, USA) to create CO_2_-enriched environment. THB (3 g THB in 100 ml dH_2_O) was used for bacterial broth cultures and experimental assays.

### Bacterial preparation

On day one, *S*. *gordonii* colonies were inoculated from the THB agar plate in 1 ml of THB in an Eppendorf tube and grown overnight at 37 °C to the stationary phase. On day two, the overnight culture of *S*. *gordonii* was diluted to a 1:5 ratio in fresh THB and incubated for another hour at 37 °C to reach log phase. The bacteria were further diluted to a 1:4 ratio in THB for the following antimicrobial efficacy assessments.

### Insert assay

**Figure 1:**
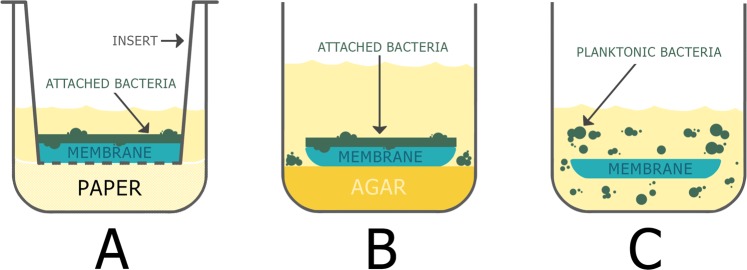
Schematic drawings of antimicrobial efficacy assays. (**A**) Insert assay; (**B**) agar contact assay; (**C**) glass-bottom well assay. The copyright owner of the drawings has granted the rights to Springer Nature Limited, for publishing the images under a CC BY open access license.

Falcon^TM^ cell culture inserts (Corning Inc. Corning, NY, USA), 0.4 µm pore size, were used as permeable supports for the ACM or PPCM membranes. Each insert was placed inside a well of 24-well tissue culture plates (Corning Inc. Corning, NY, USA). Each well contained 150 µl of THB and sterilized paper disks, which can sustain bacteria growth, underneath the insert. The membranes, ACM or PPCM, were cut into 5 mm circular pieces using a biopsy punch (Miltex, Inc., York, PA, USA) and were placed inside the inserts. *S*. *gordonii* (5 × 10^6^ cells in 40 µl volume) was spotted onto the membranes (Fig. 1A). The 24-well plates were then centrifuged at 3000 rpm for 3 minutes to ensure direct bacterial contact with the membranes, in order to better simulate bacterial adherence to the membranes in the oral cavity. After centrifuging, the plates were incubated at 37 °C for 8, 24, and 48 hours in an anaerobic culture box containing a BD BBL CO_2_ generator bag. At each time point, one of the 24-well plates was taken out of the culture box. The respective membranes were removed from the insert and placed into new wells, where the membranes were stained with 50 µl of 500X diluted (in saline) Live/Dead BacLight Bacterial Viability kit, component B (Life Technologies Corporation, Eugene, OR, USA) for 15 minutes at room temperature. The bacteria-attached sides of membranes were then placed downward in wells of the 96-well glass-bottom plate (Eppendorf North America, Hauppauge, NY, USA), and analyzed using confocal microscopy (Nikon Instrument Inc. Melville, NY, USA). For each time point—8, 24, and 48 hours of incubations, three membranes were analyzed. The experiment was repeated at least three times.

### Agar contact assay

The ACM or PPCM membranes were cut into 5 mm circular pieces using a biopsy punch and were placed on the THB agar in 96-well tissue culture plates (Corning Inc. Corning, NY, USA). Each well contained 150 µl of THB agar. *S*. *gordonii* (5 × 10^6^ cells in 40 µl volume) was spotted onto the membranes (Fig. 1B). The plates were subsequently centrifuged at 3000 rpm for 3 minutes to ensure direct bacterial contact with the membranes. After incubation at 37 °C for 8, 24, and 48 hours in the CO_2_-enriched anaerobic box, the membranes were stained in the agar wells using 50 µl of 500X diluted Live/Dead BacLight Bacterial Viability kit, component B. The bacteria-attached sides of membranes were then placed downward in wells of the 96-well glass-bottom plate, and analyzed using confocal microscopy. Three membranes to be analyzed at each incubation time point—8, 24, and 48 hours. The experiment was repeated at least three times.

### Glass-bottom well assay

*S*. *gordonii* (5 × 10^6^ cells in 40 µl THB) was added to wells in a glass-bottom 96-well plate. The 5 mm diameter ACM and PPCM membranes were subsequently placed into each well (Fig. 1C) and submerged in the planktonic bacteria. The plate was incubated for 8, 24, and 48 hours at 37 °C in an anaerobic box with a BD BBL CO_2_ generator bag. At each time point, one of the glass-bottom 96-well plates was taken out of the anaerobic box, and 40 µL of 500X diluted Live/Dead BacLight Bacterial Viability kit, component B was added to the respective wells for 15 minutes. The antimicrobial efficacy of the membranes was then analyzed using confocal microscopy. For each incubation time point—8, 24, and 48 hours respectively, three membranes were analyzed. The experiment was repeated at least three times.

### Confocal imaging

Confocal analysis was performed using a Nikon C2 plus Confocal Microscope for visual data collection, using NIS Elements AR software. Filter and laser settings utilized FITC 488 nm (Green) and TRITC 561 nm (Red). Each membrane had one or two images taken with different magnifications (100X or 200X), since the distribution of bacterial cells on the membrane was homogenous. Percentages of bacterial survival on the ACM and PPCMs were calculated as the numbers of green chains/total numbers of *S*. *gordonii* chains (green + red chains) in each of the entire images, taking advantage that *S*. *gordonii* exhibits characteristic long chains of cocci.

### Viable count recovery assays

This assay was designed to further quantify the viability of bacteria cultured on the membranes, since percentages of bacterial survival obtained by confocal analysis might be biased by the permeability of fluorescent dyes. For all the confocal assays, the membranes were removed from the wells after confocal microscopy and placed into individual Eppendorf tubes containing 500 µL of THB. The membranes and the associated bacteria were sonicated three times at setting of Output 3, Pulse, to detach *S*. *gordonii* cells from the membranes (Sonic Dismembrator Model 100, Fisher Scientific, Hampton, NH, USA). Each sample was serially diluted (10^2^, 10^4^, 10^6^) and 15 µl of each dilution was manually plated onto Mitis Salivarius Agar plates (Difco, Detroit, MI, USA). The plate was incubated at 37 °C for 24 hours in a CO_2_-enriched anaerobic jar, and colony forming units were then counted.

### Disk diffusion assays

Disk diffusion assay is a traditional method used to assess susceptibility of bacteria to antibiotics. However, this assay was used in this study to determine the diffusion ability of endogenous ACM antimicrobial components. *S*. *gordonii* in log phase (the 1-hour culture in Day 2 as described in the previous Bacteria Preparation section) were further diluted in THB to another 100X dilution, and 200 µl of which was plated on THB agar in a petri dish. A waiting period of 3 minutes was allotted to ensure the THB solution had been absorbed into the agar before proceeding to place membranes. The 5 mm ACM and PPCM membranes were soaked in saline for 5 minutes. In addition, the PPCM membranes were soaked in either tetracycline (10 mg/ml) or carbenicillin (100 mg/ml) for 5 minutes as antibiotic controls. These circular pieces were blot dried and were then placed onto the THB agar plate without dragging the membrane across the plate in order not to disrupt bacteria on the agar. The plates were cultured in an anaerobic jar with a BD BBL CO_2_ generator bag at 37 °C for 24 hours. Measurement of the bacterial clearance diameters were then performed.

### Reliability assessment

All the experiments were carried out by NP. JH independently carried out at least one experiment for every assay to confirm the reproducibility. The images taken were further reviewed by BW and CL.

### Statistical analysis

Cell counts (colony forming unit) in the viable count recovery assay at different time points were compared between the ACM and PPCM groups using the Student’s t-test. The changes of colony forming units at different time points within each group were analyzed using the one-way ANOVA, and comparisons between the means of different incubation time points were analyzed by Post Hoc Tests (Tukey). All values are expressed as mean ± standard deviation. A p-value < 0.05 was considered statistically significant.

### Institutional approval of experimental protocols

All experimental protocols in this study were approved by the UTHSC-H (University of Texas Health Science Center at Houston) Institutional Biosafety Committee. All methods were carried out in accordance with relevant guidelines and regulations. This study did not require the approval from the UTHSC-H Institutional review board for Protection of Human Subjects, since the membranes used are commercially available.

## Results

### Demonstration of antimicrobial properties in the insert assay, agar contact assay and glass-bottom well assay

**Figure 2:**
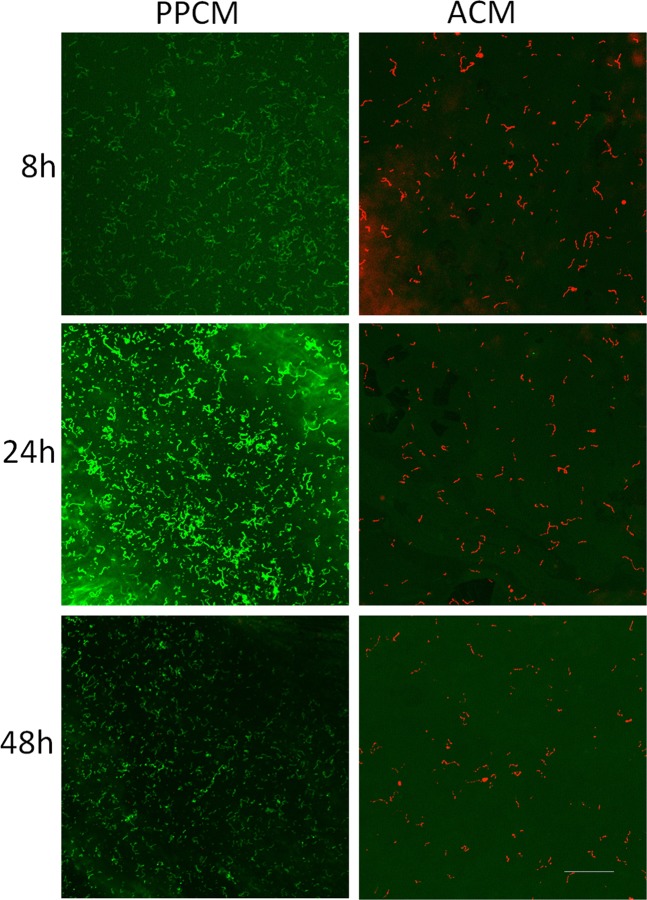
Confocal microscopy images of Insert Assays. *S*. *gordonii* incubated on PPCM and ACM at different time points were visualized after BacLight LIVE/DEAD stain. Green represents live bacteria (SYTO9), and red indicates dead bacteria (propidium iodide). Scale bar: 100 µm.

**Figure 3:**
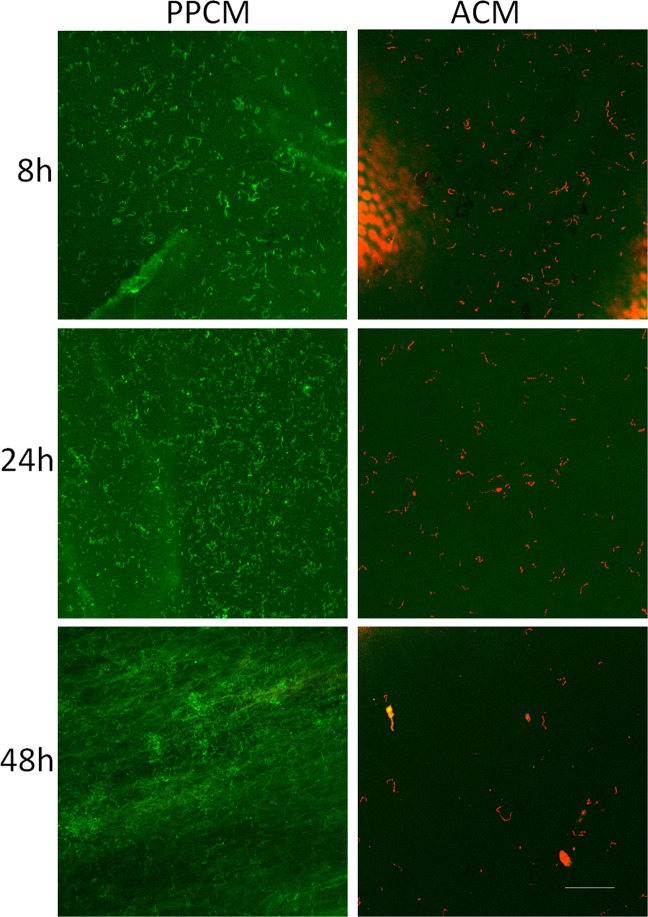
Confocal microscopy images of Agar Contact Assays. *S*. *gordonii* incubated on PPCM and ACM at different time points were visualized after BacLight LIVE/DEAD stain. Green represents live bacteria (SYTO9), and red indicates dead bacteria (propidium iodide). Scale bar: 100 µm.

**Figure 4:**
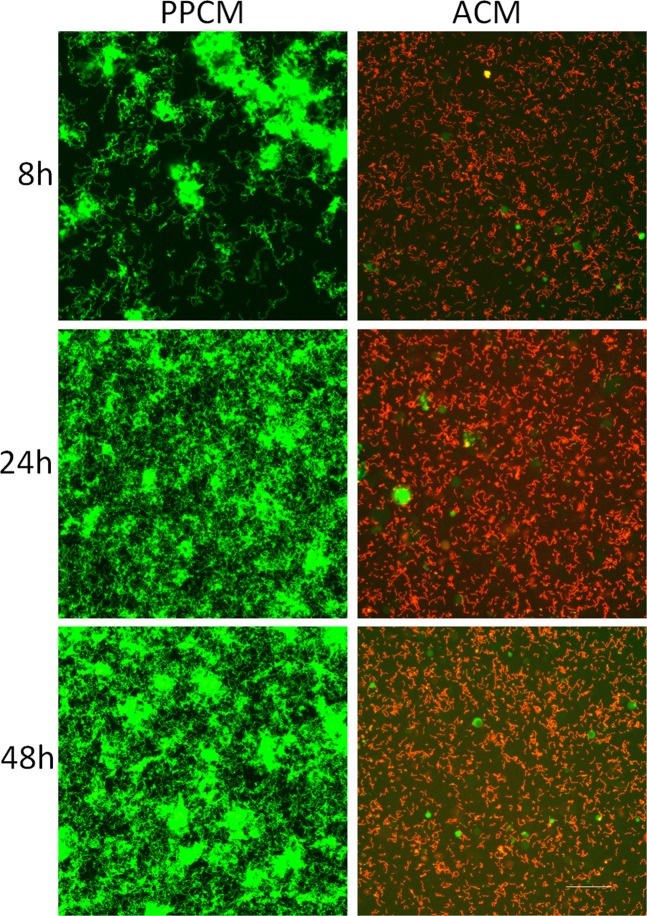
Confocal microscopy images of Glass-bottom Well Assays. Planktonic *S*. *gordonii* cells incubated with PPCM and ACM at different time point were visualized after BacLight LIVE/DEAD stain. Green represents live bacteria (SYTO9), and red indicates dead bacteria (propidium iodide). Scale bar: 100 µm.

Confocal microscopy was used to assess the viability of bacterial cells grown on the ACM and PPCMs. As shown in Figs 2–4, *S*. *gordonii* cultured on the ACM and PPCMs exhibited distinctive long chains of cocci, characteristic to streptococci, in all the assays. This allowed us to visually calculate bacterial survival rates on these membranes. All the *S*. *gordonii* chains were red in color on the ACMs, whereas >99% of them were green on the PPCMs at any time points within a 48 h testing period, indicating 0% bacterial survival on the ACMs and more than 99% survival on the PPCMs. Quantifying fluorescence intensity for bacterial cells was not performed because of the background fluorescence intensity of the membranes.

### Confirmation of bacterial death using the viable count recovery assay

**Figure 5:**
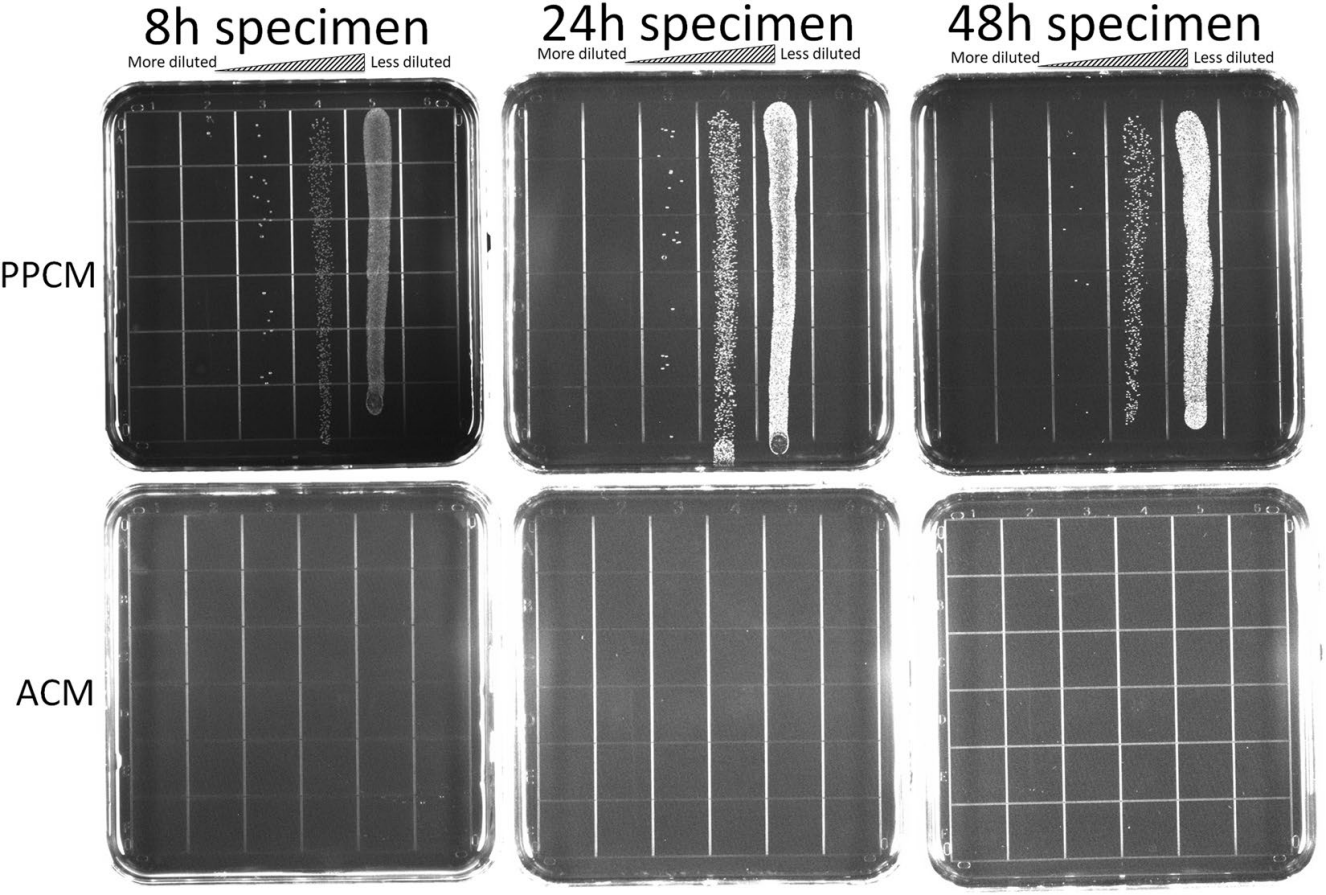
Viable count recovery assays. The upper panel: live *S*. *gordonii* on PPCM at various dilutions; the lower panel: no live *S*. *gordonii* cells on ACM at any dilutions.

There were no colony forming units (CFU, representing viable bacteria) identified on the Mitis Salivarius agar plates for the ACMs, whereas there are numerous CFUs on the plates for the PPCMs (Fig. 5). There was a significant difference in CFUs between the ACM and PPCM groups at all the time points, p = 0.0005, 0.007, and 0.0006 for 8, 24, and 48 hours of incubation, respectively.

The number of bacterial cells on the PPCMs increased from 8 to 24 hours and decreased from 24 to 48 hours (Table 1). There was a significant difference in bacterial counts among the different incubation time points (p = 0.01). Further analysis of multiple comparisons indicated that there were significant differences in cell numbers on the PPCMs between 8 h and 24 h and between 24 h and 48 h (p = 0.002 and 0.001, respectively), but not between 8 h and 48 h (p = 0.412). This assay further confirmed the results of our confocal analysis–no bacteria survived on the ACMs, whereas there was copious growth of bacteria on the PPCMs.

**Table 1:** Quantification of bacterial cells in the viable count recovery assay.

Incubation time points	cfu on PPCM	cfu on ACM
8 hours	6.52E + 06 ± 2.63E + 05	0 ± 0
24 hours	1.54E + 07* ± 2.31E + 06	0 ± 0
48 Hours	4.66E + 06 ± 1.37E + 05	0 ± 0

Colony-forming units (cfu), representing surviving *S*. *gordonii* cells, were determined after serial dilutions. Data presented are the means ± standard deviation from one of the independent experiments (triplicates for both groups). Comparisons between the means of cfu on PPCM at different incubation time points were analyzed by ANOVA Post Hoc Tests (Tukey). No surviving *S*. *gordonii* cells (0 cfu) on ACM prevented the comparisons between PPCM and ACM and within the ACM groups. * indicates significant difference in means of the 24 h group from those of the 8 h and 48 h groups on PPCM (p < 0.01).

### Demonstration of minimum diffusion ability of ACM antimicrobial components

**Figure 6:**
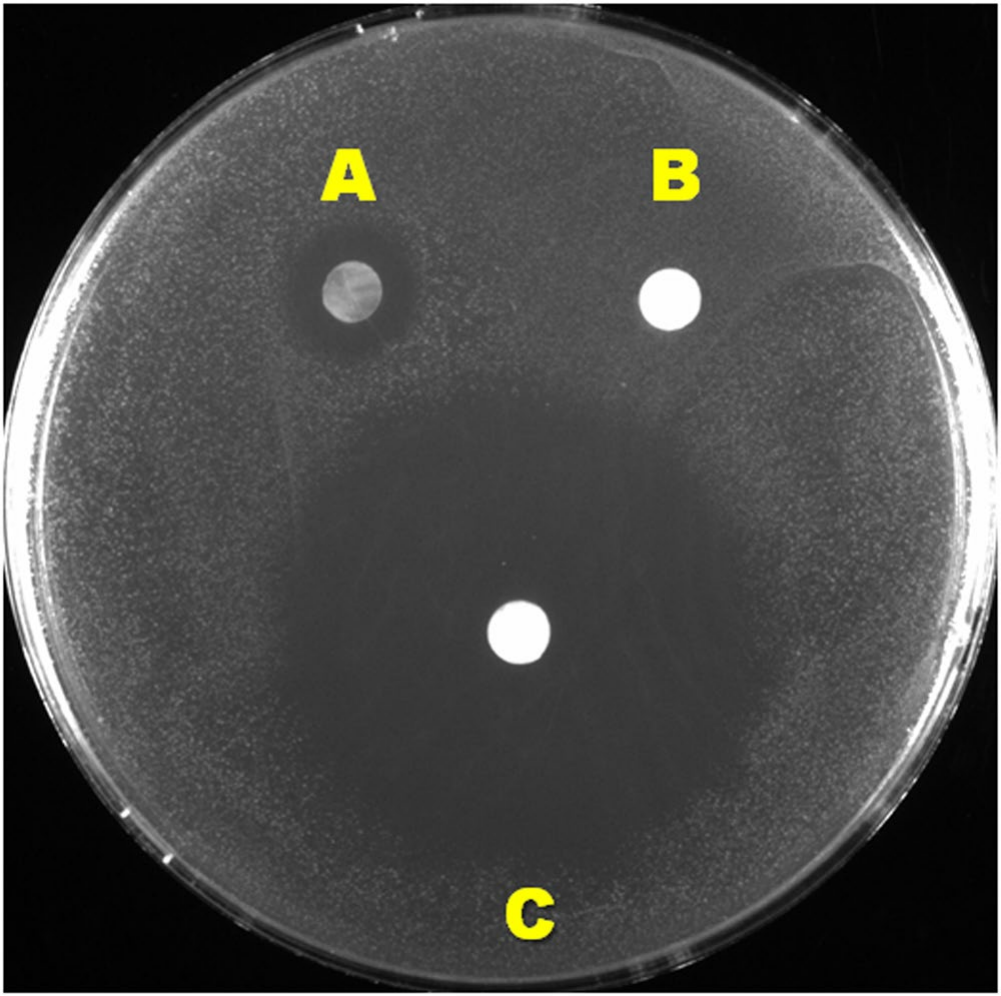
Disc diffusion assay. Log phase *S*. *gordonii* cells were plated on THB agar, and incubated with ACM (**A**), PPCM (**B**), and tetracycline-soaked PPCM (**C**) for 24 hours. The clear zone of bacterial absence around ACM was substantially smaller in diameter than that of the antibiotic disc on the agar.

The mean diameters of bacterial growth inhibition were 9.8 ± 0.8 mm for the ACMs and 41.5 ± 1.3 mm for tetracycline-soaked PPCMs, respectively. The diameter of bacterial void zone around the ACM was substantially smaller, relative to that around the tetracycline disc. There was no clear zone void of bacterial growth around the PPCM soaked with saline (Fig. 6). *S*. *gordonii* exhibited similar sensitivity toward carbenicillin (data not shown). This assay demonstrated that our target bacterium is highly sensitive to antibiotics. Since the potential antimicrobial components in the ACM are endogenous, not exogenously added antibiotics, they could not diffuse as readily as antibiotics into the agar.

## Discussion

Our main aim was to determine the efficacy of the BioXclude® membrane in preventing colonization and/or survival of oral bacteria on this collagenous membrane. However, the natural endogenous antimicrobials in BioXclude®, while still present after the dehydration process, were not shown to be readily diffusible, relative to exogenous antibiotics (Fig. 6). This prevented us from assessing its efficacy in killing oral bacteria via traditional tests such as disc diffusion assays. Hence, we designed several assays for the specific purpose of assessing the antimicrobial properties of intact membranes in conditions that mimic oral bacterial colonization in the oral cavity.

Disc diffusion assay is a traditional and common method used to test antimicrobial susceptibility of bacteria to antibiotics^24^. However, it is not a reliable method when used to test endogenous antimicrobial properties of biomaterials, such as membranes. In the disc diffusion assay, the clear zones, void of bacterial growth, around a piece of the membrane were used as indications of antimicrobial ability of the material^21^. However, this methodology did not consider the possibility of membrane shrinkage, adaptation of the membrane to the agar, or the poor diffusion ability of antimicrobial components from the membrane. Additionally, this method could only assess the absence or presence of bacterial colonies around biomaterials at a fixed incubation time^24^. The insert assay and agar contact assay, two of the three proposed assays used in the current study, enabled direct contact between the membrane and bacterial biofilm, which simulates the clinical situation in the oral cavity, whereas the glass-bottom assay, the third proposed assay in this study, reflected planktonic bacterial growth. These assays also enabled direct observation of bacterial viability on the membranes *in situ* using confocal microscopy at different incubation times, which is not possible with traditional disc diffusion assays.

Each assay had specific characteristics. Both the insert assay and the agar contact assay had the membrane stably supported by the insert or the agar in the wells, with bacterial adherence to the membrane following centrifugation. The insert assay was designed to assess the *in situ* growth of a bacterial biofilm on the membrane with sustainable fresh THB. This two-compartment culture system allows replacement of culture media without disturbing bacterial growth on the membranes. This feature will be useful in future assessment of the effect of culture conditions that reflect oral cavity variations. The agar contact assay was designed to assess the bacterial biofilm growth on the membrane in smaller amount of culture medium; agar was placed in the well to support the membrane and provide additional nutrition for bacteria, in case the nutrition in the culture medium was consumed. The glass-bottom well assay mimics planktonic growth of bacteria on the membrane. The membranes had to be moved and flipped for viability staining of bacterial cells in the insert and agar contact assays, whereas bacterial cells and the membrane could be directly stained in the glass-bottom well assay, without flipping. Flipping the membrane could disturb the bacterial cells and create technical difficulties such as enfolding of the membranes.

Each of the three assays has its advantages and limitations. The insert assay is the most technically challenging. However, the inserts can be easily moved to another well containing different culture media and is thereby suitable for assessing variations of culture conditions without disturbing the biofilm. The insert assay provides a larger amount of culture medium (a total of 190 µl, with 40 µl of THB for bacterial load and 150 µl of THB soaked in paper underneath the insert), relative to the other two assays. This larger amount of culture medium could potentially sustain a longer culture period. Since the paper underneath the insert is used to absorb most of the culture medium, there was no excessive amounts of THB covering bacterial cells on the membranes, relative to other two assays. The agar contact assay uses the THB agar in each well to support the membrane and provide additional nutrition, which is technically easier and provides a drier environment than the insert assay. The glass-bottom well assay, unlike the other two assays, allows planktonic growth of bacteria on the membranes, although biofilms can form on the membranes at later time points. It is also the easiest procedure to carry out, and the one with the least disruption to both membrane and bacteria. Taken together, these three novel assays provide *in situ* evaluation of the antimicrobial properties of these membranes at different time points, which is beyond the traditional disc diffusion assay. In addition, oral bacteria grow in both biofilm and planktonic forms in the oral cavity and environmental factors such as saliva may influence the effect of antimicrobial properties of the membrane. Each of our methods is useful in addressing different bacterial growth conditions. The insert assay is especially useful in assessing variations of the environmental factors encountered in the oral cavity, as well as for longer assessment periods than ours (48 hours) by exchanging fresh media without disturbing the biofilms on the membranes.

Relative to complete absence of live bacteria on the ACMs, there was copious growth of bacteria on the PPCMs. Biofilm bacteria on the PPCMs grew and then decreased in numbers in the agar contact assay from 8 h to 24 h and from 24 h to 48 h, respectively (Table 1). This is possibly due to either the consumption of nutrients within the small volumes of THB (40 µl/well) over the prolonged culture period, or perhaps mature biofilms shed some of their cells back to the planktonic growth phase. Given that the aim of this study was to determine the efficacy of the ACM antimicrobial properties, we did not pursue to clarify what resulted in the decrease of bacterial counts in the control PPCM membranes at 48 h. However, the results demonstrated that PPCM membranes did not exhibit any antimicrobial properties, as expected.

It is well documented that human placenta acts as a barrier against dissemination of infectious agents by a hematogenous route or by the ascending route from the vagina. There are many endogenous proteins/peptides with antimicrobial properties in the placenta^25^ and there are many natural antimicrobials present in the placenta^20,26–30^. However, no studies to date have shown whether the BioXclude® commercially available ACM, a processed and dehydrated amnion chorion collagenous membrane, exhibits antimicrobial properties. The current results demonstrate the complete elimination of tested bacterial cells on the ACM in all three assays, which suggests that this processed membrane may potentially still contain antimicrobial peptides or other antimicrobial components. Further studies are required to investigate the mechanism of action of these antibacterial properties in the ACM membrane.

*S*. *gordonii*, one of the most common oral microorganisms, and a pioneer bacterium in dental plaque, was used in these assays^31^. Although recognized as a commensal bacterium in the oral cavity, *S*. *gordonii* has been studied extensively due to its ability to promote colonization of *Porphyromonas gingivalis* (a “keystone” pathogen in periodontitis)^32^ and to cause infective endocarditis^33^. *S*. *gordonii* is detected both as biofilms in the oral cavity and as planktonic cells in saliva. It is also one of the early colonizers during dental biofilm formation. In addition, this bacterium exhibits characteristic long chains of cocci in broth or in biofilm cultures, which enables us to easily differentiate the bacterial cells from any background fluorescence in confocal analysis (Figs 2–4). Therefore, this study used *S*. *gordonii* as the target bacterium. In our confocal analysis, we were unable to estimate the number of live or dead bacteria by quantifying number of pixels due to high background fluorescence produced by the test and control membranes. However, we could distinguish clearly the red or green streptococcal chains on the ACM or PPCMs *in situ*, using confocal microscopy. The results of confocal analysis were further confirmed by a Viable Count Recovery assay. We also assessed the antimicrobial efficacy of ACM using another oral streptococcal species, *Streptococcus mutans*. Similar antimicrobial efficacies of the ACM were observed between these two oral streptococcal species (data not shown).

While bacteria flourished on the non-crosslinked control PPCM—a collagen membrane of a three-layered architecture, it should be noted that different collagen membranes have different structures and characteristics^34,35^. Thus, the current results might not be directly applied to other collagen or resorbable membranes. However, complete killing for this species by the ACM membrane in the current study demonstrated the clinical application of the ACM in the oral cavity.

Antimicrobial properties provide an advantage to the membranes used in a clinical setting given how common it is to observe membrane exposures^36^ with bacterial accumulation which leads to compromised tissue healing and regenerative potential^1,4^. Endogenous antimicrobial properties of these membranes may delay the process of membrane disintegration by eliminating oral bacteria, which secrete enzymes capable of resorbable membrane degradation^37,38^. A study demonstrated that oral bacteria grew on commonly used membranes, including collagen, expanded polytetrafluoroethylene, and polylactic acid membranes. The number of bacterial cells increased with time and none of these membranes showed notable antimicrobial properties^39^. Researchers had studied the addition of antibiotics to non-resorbable^6,7^ and resorbable membranes^8^. However, the antibiotic-impregnated membranes did not show clinical significance^40^. Studies also showed that the prescribed systemic antibiotics and chlorhexidine rinses did not effectively prevent membrane exposure^41^ and microbial contamination of the exposed membranes^42^. Antibiotics resistance was also a concern^43^. Therefore, using a membrane with antimicrobial properties, like the ACM, may improve clinical outcomes and prevent the use of antibiotics. To our knowledge, the ACM is the only collagenous membrane on the market that exhibits endogenous antimicrobial properties. ACM’s minimum diffusion ability may be beneficial in the oral cavity where the constant saliva flow might not significantly dilute the concentration of those endogenous antimicrobial components, as it does in the case of exogenously added antibiotics.

In conclusion, the human derived composite ACM membrane has high efficacy in its antibacterial activities. The assays we utilized in this study served our specific aim of testing endogenous antimicrobial properties of intact membranes in conditions that mimic oral bacterial colonization in the oral cavity. These assays could be used to test the antimicrobial efficacy of other biomaterials with minimum diffusion ability in the future.

## Data Availability

All data are available upon request. Materials used in the study are commercially available.
